# Supplementary material for: Spatial EGFR Dynamics and Metastatic Phenotypes Modulated by Upregulated EphB2 and Src Pathways in Advanced Prostate Cancer
Source: Cancers (Basel). 2019 Dec 1;11(12):1910. doi: 10.3390/cancers11121910 (PMC6966510; doi:10.3390/cancers11121910)
Supplement: Supplementary file 1 [file cancers-11-01910-s001.pdf]

Supplementary material

Spatial EGFR dynamics and metastatic phenotypes modulated by upregulated EPHB2 and SRC pathways in advanced prostate cancer

Yen-Liang Liu[1,2,†], Aaron M. Horning[3,†], Brandon Lieberman[4], Che-Kuang Lin[3], Chia-Nung Hung[3], Chih-Wei Chou[3], Chiou-Miin Wang[3], Chun-Lin Lin[3], Nameer B. Kirma[3], Michael A. Liss[5], Mirae Kim[2], Rohan Vasisht[2], Evan P. Perillo[2], Katherine Blocher[2], Hannah Horng[6], Josephine A. Taverna[7], Jianhua Ruan[8],Thomas E. Yankeelov[2,9,10,11,12], Andrew K. Dunn[2], Tim H.-M. Huang[3], Hsin-Chih Yeh[2,13,*] & Chun-Liang Chen[3,*]

[1]Graduate Institute of Biomedical Sciences, China Medical University, Taichung, Taiwan

[2]Department of Biomedical Engineering, University of Texas at Austin, Austin, TX, USA

[3]Department of Molecular Medicine, Mays Cancer Center, University of Texas Health Science Center, San Antonio, TX, USA

[4]Department of Biology, Trinity University, San Antonio, TX, USA

[5]Department of Urology, University of Texas Health Science Center, San Antonio, TX, USA

[6]Department of Bioengineering, the University of Maryland, College Park, MD, USA

[7]Department of Medicine, Mays Cancer Center, University of Texas Health Science Center, San Antonio, TX, USA

[8]Department of Computer Science, University of Texas at San Antonio, TX, USA

[9]Institute for Computational Engineering and Sciences, University of Texas, Austin, TX, USA

[10]Department of Diagnostic Medicine, Dell Medical School, University of Texas at Austin, Austin, TX, USA

[11]Department of Oncology, Dell Medical School, University of Texas at Austin, Austin, TX, USA

[12]Livestrong Cancer Institutes, University of Texas at Austin, Austin, TX, USA

[13]Texas Materials Institute, University of Texas at Austin, Austin, TX, USA

Contents

Method S1 | 2D single-particle tracking 1
Method S2 | Extracting dynamic parameters from MSD 2
Method S3 | 3D single-particle tracking 2
Method S4 | Two-photon laser scanning microscope (2P-LSM) 3
Method S5 | Data processing for 3D single-particle tracking and 2P scanning imaging 4
Method S6 | 3D Inward movement analysis 4
Figure S1 | Characterization of EGFR diffusivity and compartment size 6
Figure S2 | Evaluation of EGFR internalization by a 3D single-particle tracking technique 7
Figure S3 | Process of quantification of cortical actin on the apical side of the plasma membrane 8
Figure S4 | Quantification of cortical actin on the apical side of the plasma membrane in Lat-B treated cells 9
Figure S5 | Unsupervised hierarchical clustering of DE gene expression in EPHB-mediated forward signaling. 11
Figure S6 | The transcript expression of actin-binding protein genes downstream to Rho GTPases and their associated effectors in PC cells. 12
Figure S7 | Eph-ephrin and Src signaling expression profile in prostate circulating tumor cells. 13
Figure S8 | Upregulation of DE genes in Src and EphB2 pathways predicts high grades and poor disease-free survival in TCGA prostate cancer patient cohort. 14
Figure S9 | Dasatinib attenuated cell motility, invasion and EGFR diffusion in PC3. 15
Figure S10 | Quantification of cortical actin on the apical side of the plasma membrane in gene-knocked down cells. 16
Figure S11 | Knockdowns of SRC and EPHB2 mRNA affect migration and invasion. 17
Figure S12 | EGFR expression of prostate cancer cell lines 18
Figure S13 | Westernblots 19
Table S1 | Differentially expressed genes of EPH, EPHB forward, and SRC pathways in PC3 20
Table S2 | The qRT-PCR primer sequences for validation of DE gene expression 27
Table S3 | Summary of patients' pathological status and CTCs collected. 29
Supplementary Reference 30

Method S1 | 2D single-particle tracking

Single-particle trajectories were determined from the raw data sets using a three-step process: (i) Identifying contiguous regions of pixels; (ii) Gaussian fitting; (iii) building trajectories from coordinates. This approach is similar to those described previously[1-3].

1. Identifying contiguous regions of pixels. A series of 2D images in time trace was processed independently to find FN-IgG-EGFR coordinates. The contiguous regions of pixels, which represent the images of fluorescent particles, were identified on the basis of two criteria: (i) pixels had intensities greater than 3-fold the standard deviation of pixel intensities from areas defined as background (background offset algorithm[4]) and (ii) pixels were above a threshold[4]. Then, a high pass filtering was applied to the image with a 2D Gaussian filter (σ = 5). The binary image of pixels passing both criteria was later processed by Gaussian fitting.

2. Gaussian fitting. To find out the center of the fluorescent particles, the center of mass of each contiguous region in the binary image was set as the starting point in a Gaussian fitting routine. The highest intensity pixel in a small region around the starting point (5 pixels square) was used as an updated starting. Fits were performed in a square region, of size ~ 2*σ_{psf}, around the updated starting point. The σ_{psf} defines the size of 2D Gaussian approximation to the point spread function. After convergence of the fitting routine (a change in location of fewer than 10^{-5} pixels), a normalized cross-correlation was calculated to verify the 2D Gaussian-fitted coordinates. The found coordinates were only considered as positions of FN-IgG-EGFR and used in the further analysis if they exceeded a cross-correlation value of 0.7.

3. Building trajectories from coordinates. The probability of finding a diffusing particle with diffusivity *D* in two dimensions at a distance greater than *d* from its starting point after a time *Δt* is given by[5]

$$P(d, \Delta t) = exp\left[\frac{-d^2}{4D\Delta t}\right] \qquad (1)$$

Trajectories were built from the set of 3D coordinates (x,y, and t) in two steps. First, coordinates identified at time *t* were compared with coordinates at time *t+Δt* using Eq. 1 where *Δt* is the inverse frame rate of data acquisition. If *P(r, Δt)* was found to be greater than .05, the coordinate at *t+Δt* is associated with the coordinate at t in a trajectory. This process builds short, un-interrupted trajectories. Second, to connect these short trajectories originated from the same targets, the end coordinate of all trajectories is compared with all later starting coordinates of other trajectories using Eq. 1, where *Δt* is now the time interval between the end of the first trajectory and the beginning of the second. The later trajectory with the smallest *Δt* that has a *P(r, Δt)*> 0.01 is connected with the first trajectory. This process is continued

until there are no remaining pairs of trajectories that satisfy the criteria. The reconstructed trajectories are further processed into mean-squared displacement to estimate diffusion coefficient.

Method S2 | Extracting dynamic parameters from MSD

The typical approach to analyze a single-particle trajectory starts with the calculation of mean-squared displacement (MSD)[6, 7], which describes the average squared distance (d^2, r is the position vector) that the particle has explored in space at a given time lag (Δt):

$$MSD(\Delta t) = \langle (\boldsymbol{r}(t + \Delta t) - \boldsymbol{r}(t))^2 \rangle \qquad (2)$$

Collected trajectories all started on the apical surfaces of the cells and exhibited an interchange of confined diffusion and Brownian diffusion. The dynamics parameters, the diffusivity (D) and the linear dimension of compartments (L), are extracted from trajectories by fitting the MSD curves. The D was defined as the linear MSD fitting result of the first 5 MSD points:

$$MSD(\Delta t) = \langle (\boldsymbol{r}(t + \Delta t) - \boldsymbol{r}(t))^2 \rangle = 4D_{1-5}\Delta t \qquad (3)$$

For L, the MSD curves were fitted with an equation for confined diffusion[8, 9], and L was defined as the MSD fitting result of the first 10 MSD points:

$$MSD(\Delta t) \cong \frac{L^2}{3}\left[1 - \exp\left(-\frac{\Delta t}{\tau}\right)\right] + 4D_{macro}\Delta t \qquad (4)$$

Confined diffusion is featured by an abrupt change of slope in the MSD curve[10] after a characteristic equilibration time τ.

Method S3 | 3D single-particle tracking

TSUNAMI (Tracking of Single particles Using Nonlinear And Multiplexed Illumination) is a feedback-control tracking system which employs a spatiotemporally multiplexed two-photon excitation and temporally demultiplexed detection scheme. Sub-millisecond temporal resolution (under high signal-to-noise conditions) and sub-diffraction tracking precision in all three dimensions have been previously demonstrated[11-13]. Tracking can be performed in a live cell to monitor the movements of fluorescent nanoparticle-tagged EGFRs[13, 14] or ballistically injected fluorescent nanoparticles[15]. The TSUNAMI microscope has been described in detail in our previous study 10. In brief, excitation of 800 nm from a Ti:Al_2O_3 laser (Mira 900, Coherent) was used for tracking experiments. The optical multiplexer splits the primary laser beam into 4 beams which then passed through a galvo scanning system (6125H, Cambridge Technology). These 4 beams were focused through a 60× N.A. 1.3 silicone oil objective (UPLSAPO 60X, Olympus) to create a tetrahedral excitation profile. The total laser power was ~8mW at the objective back aperture. The photon count rates of a typical ⌀40 nm red fluorescent bead (F8770, Thermo Fisher Scientific) were in the range of 200-500 kHz. Signal-to-noise ratios were typically above 20 with 3 kHz background fluorescence signal and 150 Hz background signal from the detector. Temporally demultiplexed detection was performed via time-correlated single-photon counting (TCSPC)

analysis. Fluorescence signals were detected by a cooled GaAsP photomultiplier tube (PMT) with 5 mm square active area (H7422PA-40, Hamamatsu) in the non-descanned configuration. The current output from the PMT was amplified through a 2 GHz cutoff bandwidth preamplifier (HFAC-26, Becker and Hickl GmbH) and sent into a photon-counting board (SPC-150, Becker and Hickl GmbH) to be counted and correlated to the 76 MHz reference clock of the laser oscillator. The instrument response function (IRF) was measured to be 230 ps FWHM. Every 5 ms a photon histogram was sampled from the TCSPC module and processed in the software loop run in LabVIEW (National Instruments). The tracking algorithm employs a proportional control to convert the error signals to new stage positions. New voltages were sent out through a DAQ card (PCIe-6353, National Instruments) to their respective actuators – galvo mirrors for x and y tracking and piezo objective stage for z tracking. The saved voltages were converted to build a 3D tracking trajectory.

Method S4 | Two-photon laser scanning microscope (2P-LSM)

The excitation light for laser scanning imaging shared the same path as the non-delayed beam in the multiplexer, the other 3 beams were blocked with shutters during laser scanning. By the nature of 2P excitation, excitation of 800 nm from a Ti:Al_2O_3 laser (Mira 900, Coherent) is able to excite Hochest 33258 (Hochest 33258, H3569, Thermo Fisher Scientific), red fluorescence nanoparticle (F8770, Thermo Fisher Scientific), and Cellmask™ Deep Red (C10046, Thermo Fisher Scientific). The emission of these three fluorophores was collected simultaneously with three individual photomultiplier tubes (PMT).

The excitation light for laser scanning imaging shared the same path as the non-delayed beam in the multiplexer. The emission light was collected through the same objective and transmitted through the dichroic filter to be condensed onto three PMTs. The emission light was split into three color bands by two dichroic mirrors before being condensed onto each PMT (H7422PA-40, Hamamatsu). The first dichroic mirror picks of the blue-green spectrum with a reflection edge at 550 nm (FF552-Di02-25x36, Semrock) the second dichroic splits the remaining light into orange and red components with an edge at 640nm (FF640-FDi01- 25x36, Semrock). Each PMT has a bandpass filter specific to the fluorescent targets used for the experiment, 460-500nm for nuclear stain (Hochest 33258, H3569, Thermo Fisher Scientific), 570-610nm for red fluorescent nanoparticle (F8770, Thermo Fisher Scientific) and 680-720nm for membrane stain (Cellmask™ Deep Red, C10046, Thermo Fisher Scientific). The mirror scanning and data acquisition were handled by a single DAQ card (PCIe-6353, National Instruments) with a custom code interface in LabVIEW. Pixel dwell times were 2.6 microseconds which correspond to a frame rate of approximately 1.4 Hz. Image sizes were typically 51 × 51 μm^2.

Method S5 | Data processing for 3D single-particle tracking and 2P scanning imaging

All data processing was performed in MATLAB (Mathworks). Saved in a binary format, the trajectory raw data contained photon counts and voltage outputs from the actuators (i.e. the xy scanning galvo mirrors (6125H, Cambridge Technology) and the objective z-piezo stage (P-726 PIFOC, PI)) at each 5 ms time point. Trajectories were plotted by simply connecting particle positions of consecutive time points. 2P-LSM raw images were read into MATLAB from binary files and denoised with a median filter before a 1D interpolation along the z dimension. To segment the cellular compartments, we used a simple intensity threshold technique that converts the image to a binary. Thresholds were selected at each z plane to account for variation in noise and brightness through the z-stack. The binary images were used as a mask to plot the cell isocontour. Because trajectories were measured with the same analog output device as the 2P-LSM images they can be directly overlaid with the cell compartment isocontour with no conversion or scaling required.

Method S6 | 3D Inward movement analysis

The 3D inward movement analysis is adapted from Picco's 2D inward movement analysis[16], and it has been used to analyze the anti-PDL1 antibody-induced PD-1 internalization[17]. The 3D inward movement analysis is a two-step procedure based on the 2P scanning imaging and 3D single-particle tracking. Firstly, the 3D-stacked live-cell image was acquired by 2P-LSM. Secondly, the fluorescently labeled EGFR was tracked by the TSUNAMI microscope. Then the EGFR trajectory was coregistered with the 3D cell image. The distance and velocity of the inward movement were then derived from the collected trajectories.

The 3D inward movement analysis is a two-step procedure based on the 2P scanning imaging and 3D single-particle tracking. Firstly, the 3D-stacked live-cell image was acquired by 2P-LSM. Secondly, the fluorescently labeled EGFR was tracked by the TSUNAMI microscope. Then the EGFR trajectory was coregistered with the 3D cell image. The distance and velocity of the inward movement were then derived from the collected trajectories. To visualize the plasma membrane and nuclei, cells were stained with CellMask™ Deep Red and Hoechst 33258. To define the orientation of inward movement, a tangent plane on the plasma membrane was derived using the start point of a trajectory and the reconstructed 3D isocontour of the plasma membrane. The normal vector which is perpendicular to the tangent plane is the axis of inward movement. Then the trajectory was projected to the normal vector, and the displacement along the normal vector is defined as the inward movement. For sample preparation, cells were seeded onto optical imaging 8-well chambered coverglass (155409, Thermo Fisher Scientific) with the cell density of 1×10^5 cells per well and allowed to grow to ~70% cell confluency. Before tracking experiments, cells were stained with a mixture of Hoechst 33258 (H3569, Thermo Fisher Scientific,

1:1000 dilution in complete medium) and CellMask™ Deep Red (C10046, Thermo Fisher Scientific, 1:1000 dilution in complete medium) for 10 minutes at 37ºC. After membrane staining, the staining buffer was replaced with the antibody solution (antibody-conjugated fluorescent nanoparticles at 100 pM). The reaction was incubated for 10 minutes at 37ºC and the antibody solution was removed, subsequently. Sample cells were washed twice using PBS to remove the unbound fluorescent nanoparticles. Upon completion of membrane staining and antibody labeling, the chambered coverglass was immediately placed on the TSUNAMI microscope for tracking experiments. Two to four trajectories (duration ranged from 1-10 minutes) were typically obtained from each well after the EGF stimulation (20 ng/mL EGF (recombinant human epidermal growth factor, PHG0311L, Thermo Fisher Scientific). The volumes of all solutions and washing buffers used in staining were 200 μl per well. All data processing was performed in MATLAB (Mathworks) and saved in a binary format. The trajectory raw data contained photon counts and voltage outputs from the actuators (i.e. the xy scanning galvo mirrors (6125H, Cambridge Technology) and the objective z-piezo stage (P-726 PIFOC, PI)) at each 5 ms time point. Trajectories were plotted by simply connecting particle positions of consecutive time points.

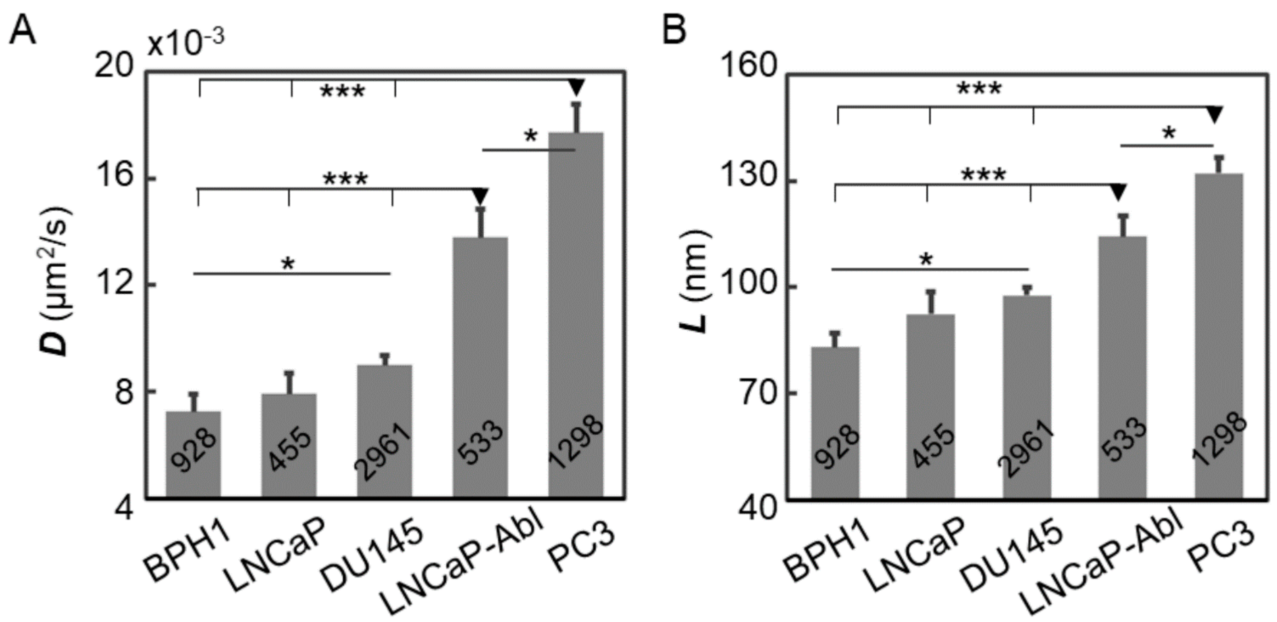

Figure S1 | Characterization of EGFR diffusivity and compartment size

A panel of five prostate cell lines was evluated with the TReD assay to characterize their EGFR diffusivity (*D*) and compartment size (*L*). There is a trend of increased EGFR diffusivities and compartment sizes associated with advanced malignancy. PC3 exhibited significantly the highest diffusivity and largest compartment size. The number of trajectories collected from each cell line is shown on each bar. Statistical comparison was performed using unpaired t-test., where the asterisk represents statistical significance: ***p<0.001, **p<0.01, *p<0.05. The error bar represents the standard error of the mean.

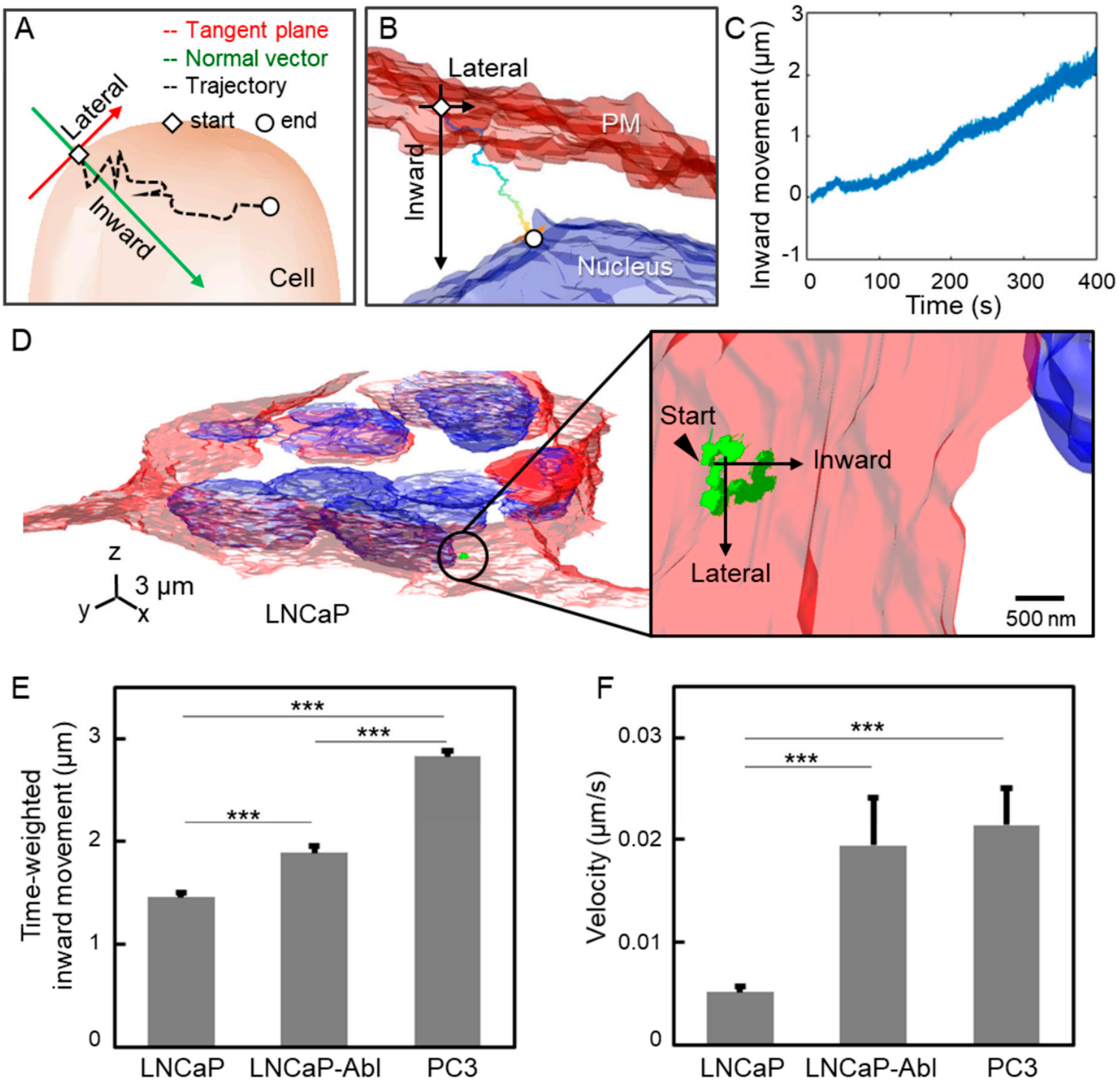

Figure S2 | Evaluation of EGFR internalization by a 3D single-particle tracking technique

(A) The schematic explains that the direction of inward movement is defined as the normal vector of the tangent plane on the plasma membrane at the start point of a trajectory. Then the trajectory was projected on the normal vector to derive the inward movement. **(B)** A representative trajectory demonstrates the internalization of an EGFR in an A431 cell whose plasma membrane (PM) and nucleus were stained with CellMask Deep Red and Hoechst, respective. The inward movement of the trajectory is plotted in **(C)**. **(D)** A 2P-scanning image of LNCaP cell and the inward movement of the EGFR into the cell. **(E), (F)** EGFR internalization was characterized by the trajectory-derived parameters: inward movement and velocity. The error bar represents the standard error of the mean from 9-20 measurements. All statistical analysis was performed using the unpaired t-test. The asterisk represents the level of statistical significance for t-test: *** $p < 0.001$.

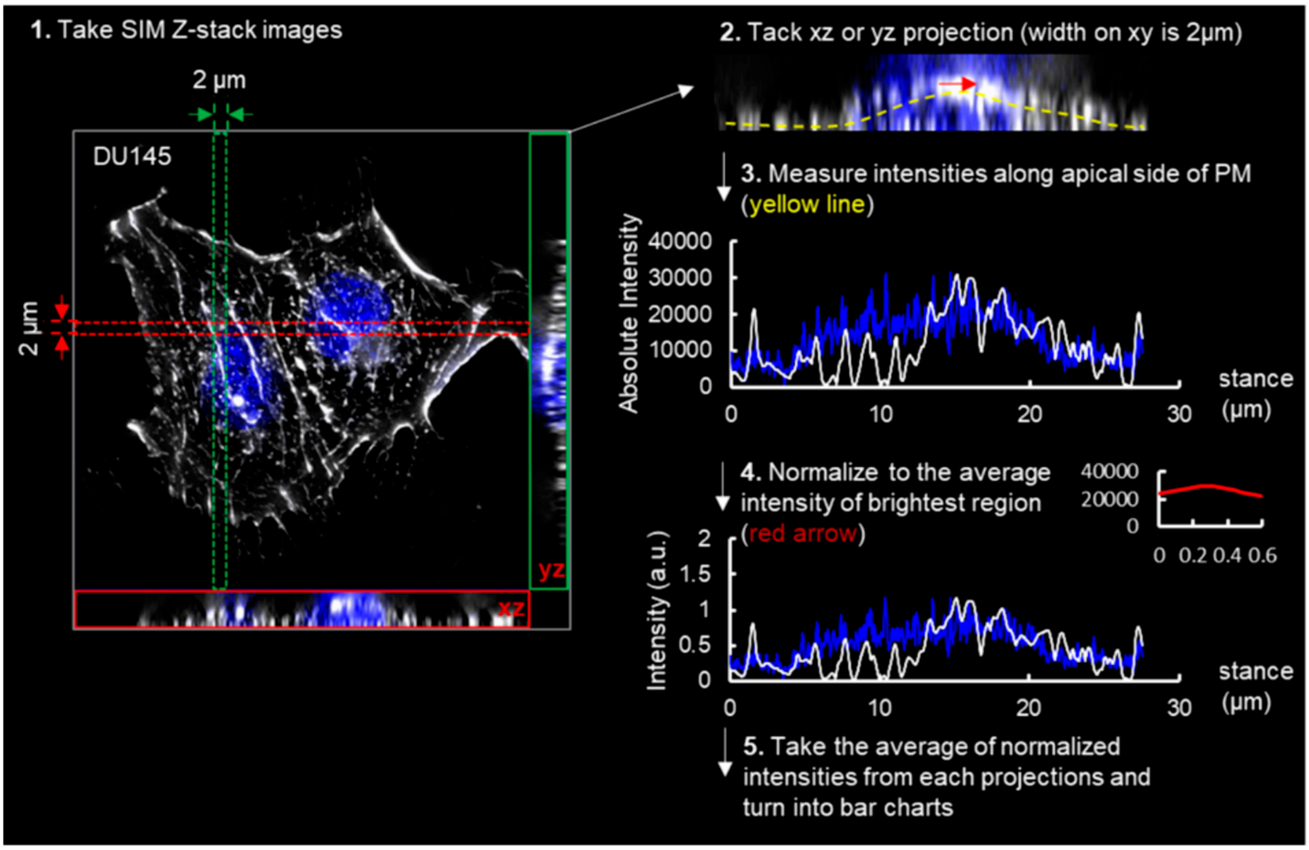

Figure S3 | Process of quantification of cortical actin on the apical side of the plasma membrane
The quantification of the fluorescence intensity of cortical actin was conducted using the software: ZEN 2.3 lite. The xz and yz projections of 2 µm width on the xy plan were extracted from the Z-stack of SR-SIM images. The fluorescence intensity of actin along the apical side of the plasma membrane and the brightest region were measured. To make a fair comparison among batches of images, the measured intensities were normalized with the average intensity of brightest region in the same projection. The average of the normalized fluorescence intensity of actin was taken as an indicator of the density of cortical actin. The procedure of image-based quantification of cortical actin was summarized below, and more representative fluorescence profiles were presented in **Figure S4** and **Figure S10.**

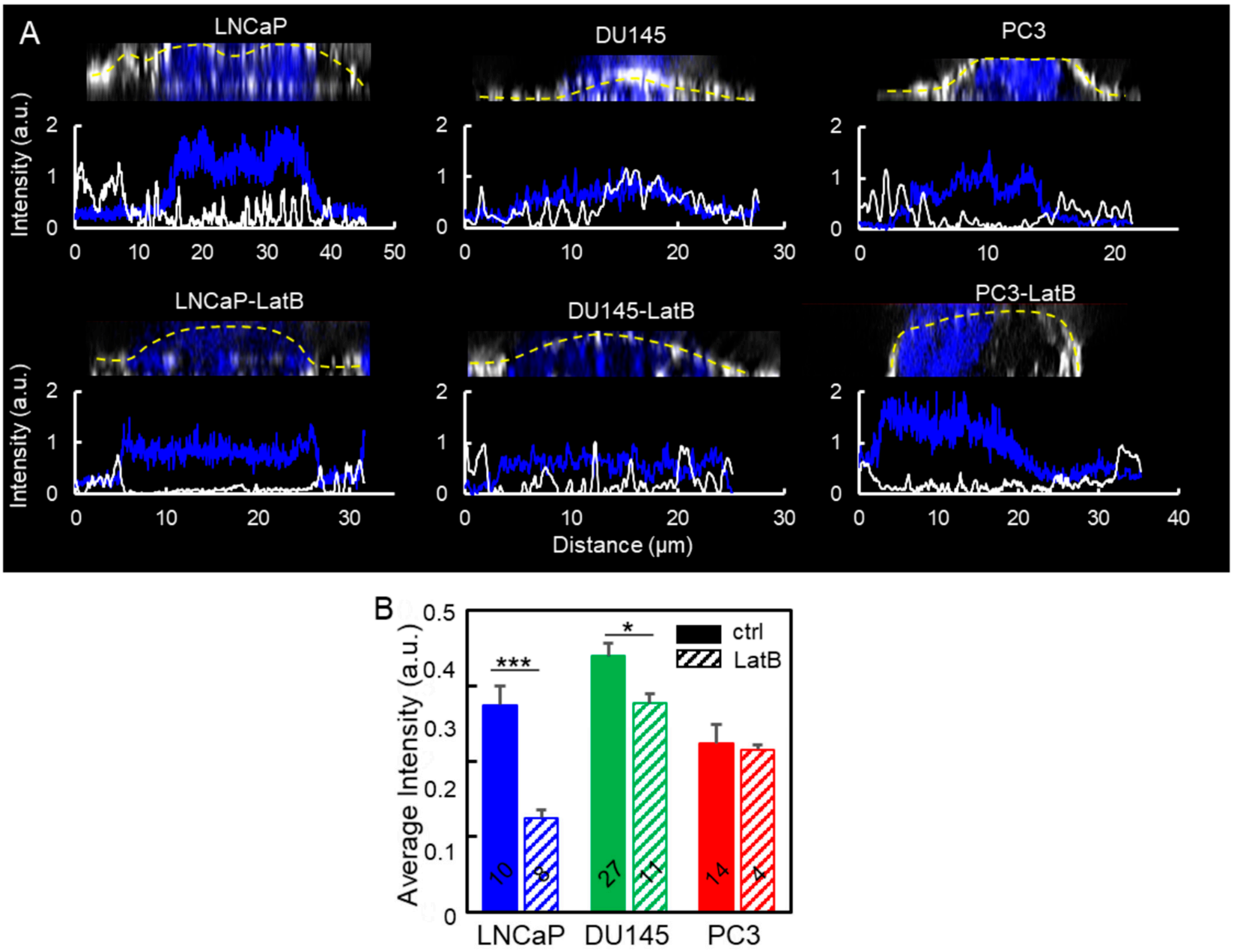

Figure S4 | Quantification of cortical actin on the apical side of the plasma membrane in Lat-B treated cells

(A) The representative images of xz projections of cells with fluorescently labeled actin filaments (white) and nuclei (blue), which are also shown in **Figure 2**. The corresponding profiles of the normalized fluorescence intensity along the apical side of the plasma membrane indicated by yellow dashed lines. The fluorescence intensities are normalized and presented as an arbitrary unit (a.u.). **(B)** Quantification of cortical actin-based on the measured fluorescence intensities. The averages of normalized intensities were presented, and the number of projections analyzed is labeled on each bar. All statistical analysis was performed using the unpaired t-test. The asterisk represents the level of statistical significance for t-test: * $p < 0.05$, *** $p < 0.001$. The error bar represents the standard error of the mean.

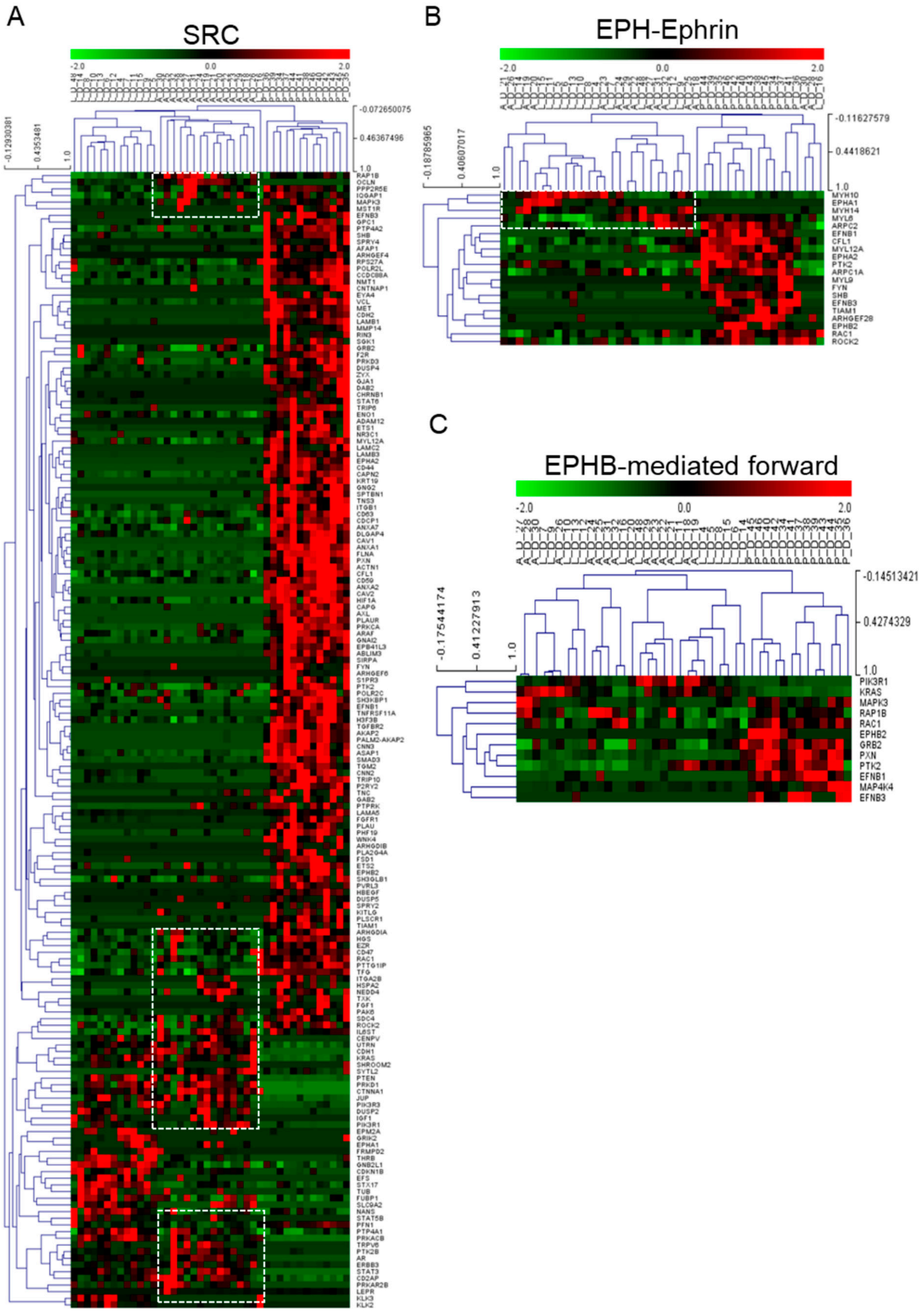
A
SRC
-2.0
0.0
2.0
-0.072650075
0.46367496
1.0
-0.12930381
0.4353481
1.0
B
EPH-Ephrin
-2.0
0.0
2.0
-0.11627579
0.4418621
1.0
-0.18785965
0.40607017
1.0
MYH10
EPHA1
MYH14
MYL6
ARPC2
EFNB1
CFL1
MYL12A
EPHA2
PTK2
ARPC1A
MYL9
FYN
SHB
EFNB3
TIAM1
ARHGEF28
EPHB2
RAC1
ROCK2
C
EPHB-mediated forward
-2.0
0.0
2.0
-0.14513421
0.4274329
1.0
-0.17544174
0.41227913
1.0
PIK3R1
KRAS
MAPK3
RAP1B
RAC1
EPHB2
GRB2
PXN
PTK2
EFNB1
MAP4K4
EFNB3

Figure S5 | Unsupervised hierarchical clustering of DE gene expression in EPHB-mediated forward signaling.

(A) Unsupervised hierarchical clustering of differentially expressed (DE) genes (171) in Src signaling. LNCaP (LN), LNCaP-Abl (ABL) and PC3 are distinct clusters separated from the other clusters. **(B)** Unsupervised hierarchical clustering of DE gene expression in Eph-ephrin signaling. The dashed white lines delineate the gene sets upregulated in LNCaP-Abl cells. **(C)** ScRNA-seq showed that the majority of genes in the EPHB-mediated forward pathway are upregulated in PC3 cells. LNCaP and LNCaP-Abl cells display heterogeneity but are clustered together based on DE genes in EPH-Epherin and EPHB-mediated signaling.

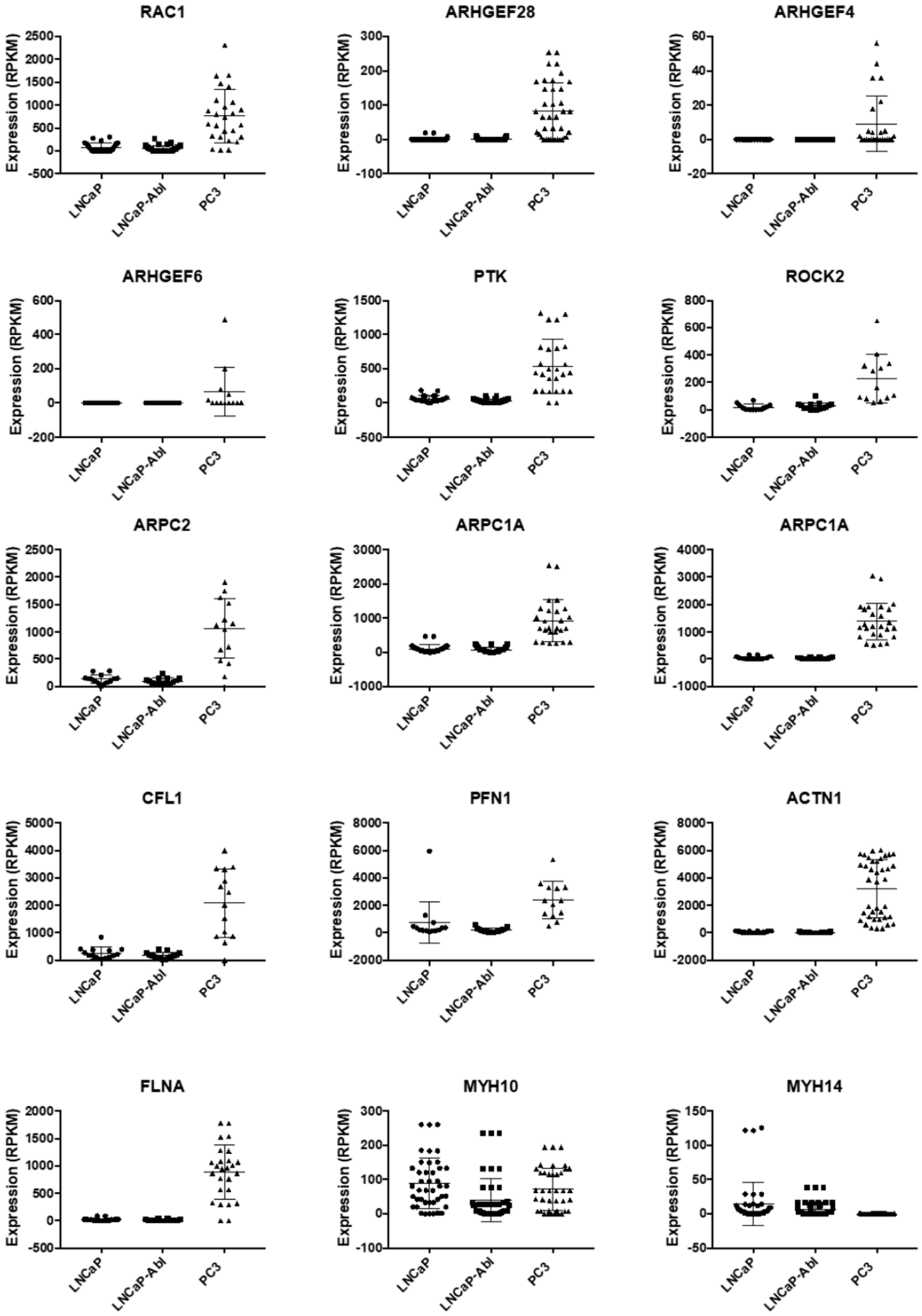

Figure S6 | The transcript expression of actin-binding protein genes downstream to Rho GTPases and their associated effectors in PC cells.

The transcript expression of genes in actin-binding proteins and regulators are based on scRNA-seq data and shown in RPKM. Most of the gene transcripts are upregulated in more aggressive PC3 cells. The transcript expression of genes in actin-binding proteins and regulators are based on scRNA-seq data and shown in RPKM.

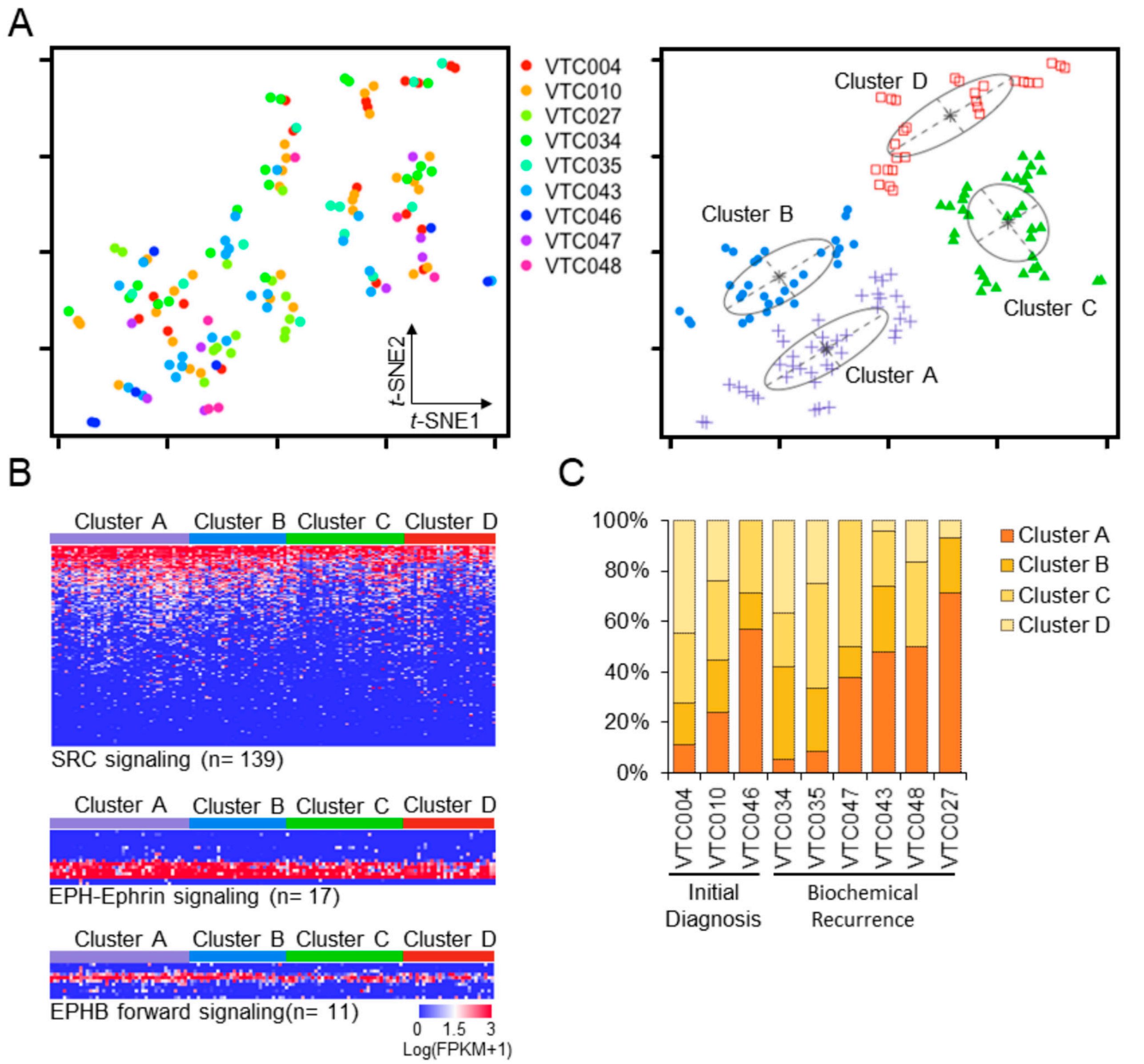

Figure S7 | Eph-ephrin and Src signaling expression profile in prostate circulating tumor cells. **(A)** t-Distributed Stochastic Neighbor Embedding (t-SNE) scatter plot of nine prostate cancer patients CTCs based on SRC and EphB/ephrinB signaling expression profile. **(B)** Four different clusters were identified and projected in t-SNE scatter plot. **(C)** Proportion of the CTCs in each cluster per patient.

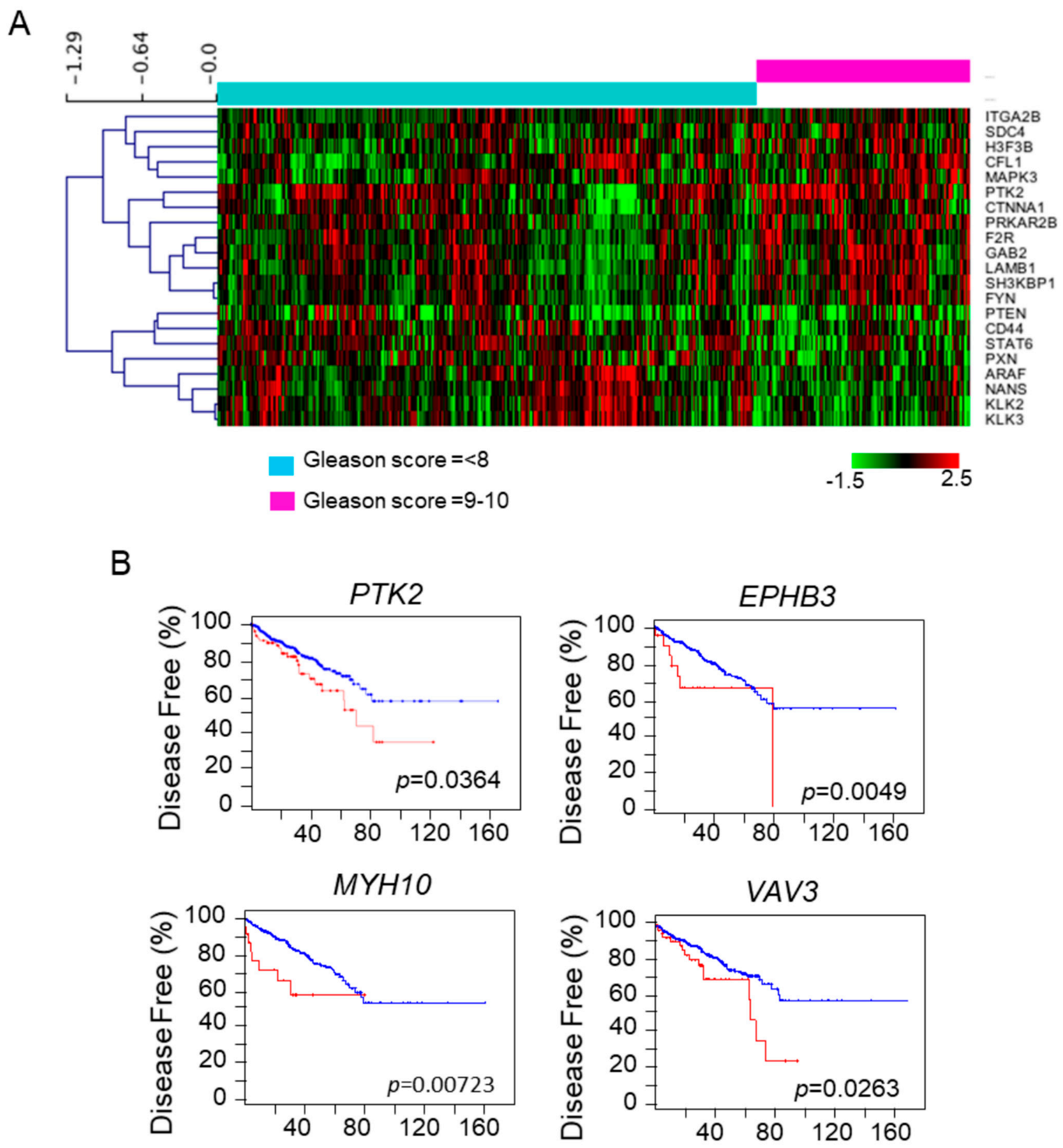

Figure S8 | Upregulation of DE genes in Src and EphB2 pathways predicts high grades and poor disease-free survival in TCGA prostate cancer patient cohort.

(A) Expression pattern of 21 genes differentiates high-grade tumors with Gleason score 9-10 from tumors with Gleason score ≤ 8. **(B)** The alternation of four additional genes predicts poor prognosis using Kaplan-Meier estimation analysis.

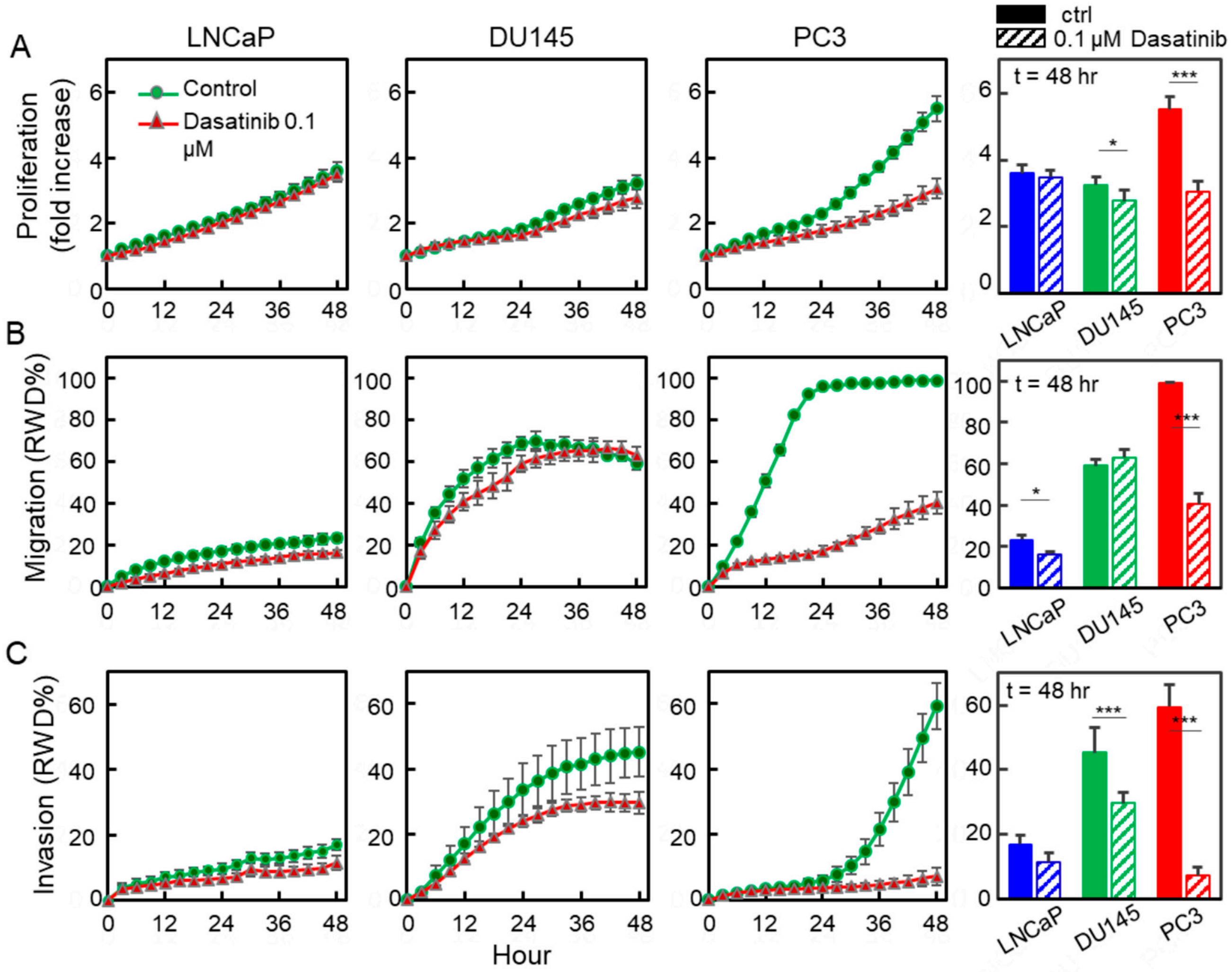

Figure S9 | Dasatinib attenuated cell motility, invasion and EGFR diffusion in PC3.

(A)-(C) The attenuation of proliferation, migration, invasion of three PC cells (LNCaP, DU145, and PC3) treated with dasatinib, an inhibitor to EPHB/SRC pathways. The image-based IncuCyte assays allow us to conduct the time-lapse analysis of cell proliferation, migration, and invasion of the cells treated with or without dasatinib. The dasatinib significantly inhibits the proliferation, migration, and invasion of the highly metastatic prostate cancer cells (PC3). The dasatinib also significantly decreases the EGFR diffusivity in PC3 cells. The mean value of each bar was measured at the end time of each assay or at 48th-hour. All statistical analysis was performed using the unpaired t-test. The asterisk represents the level of statistical significance for t-test: *** $p < 0.001$, ** $p < 0.01$, * $p < 0.05$. The error bar represents the standard error of the mean. The RWD stands for relative wound density.

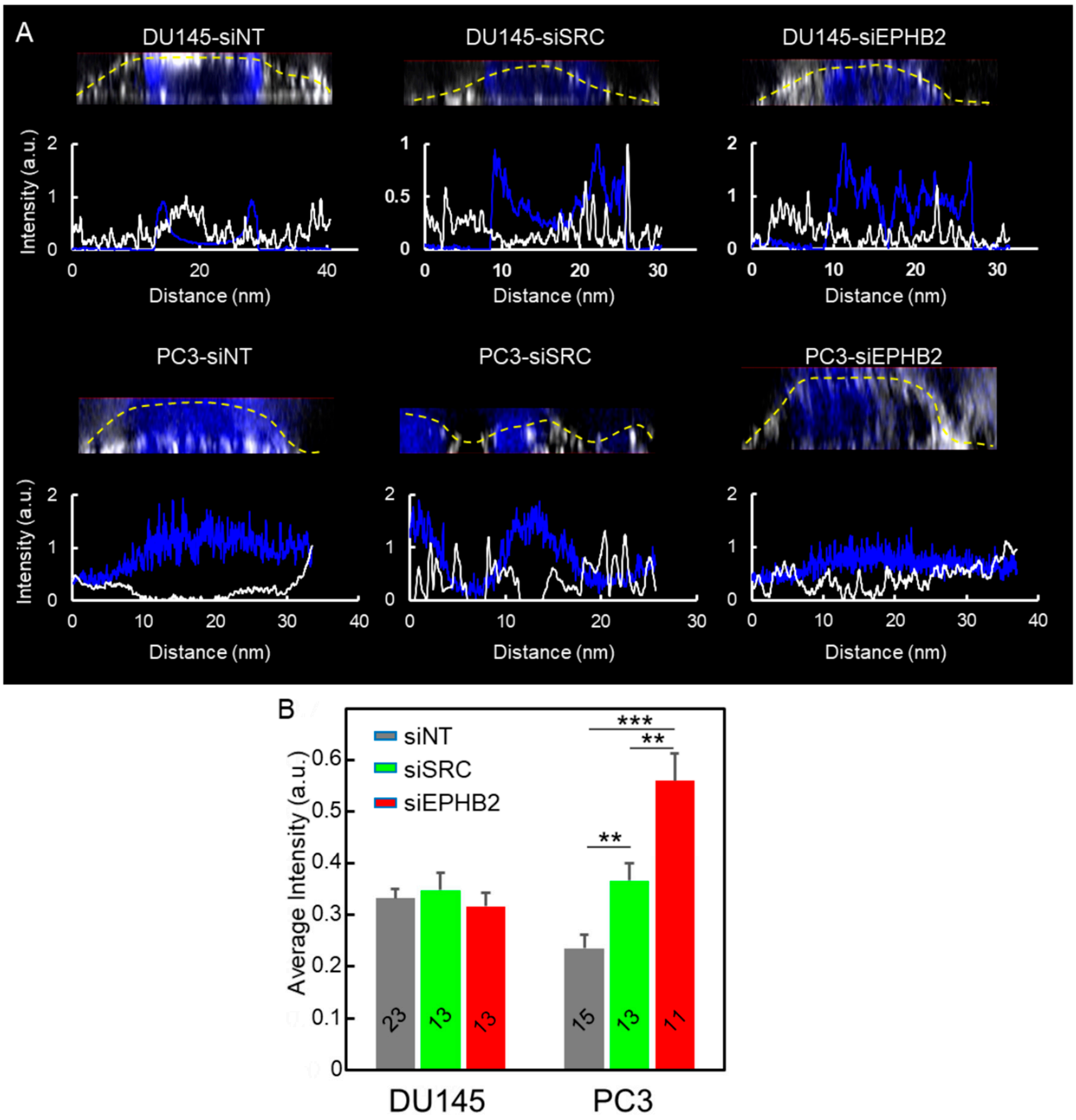

Figure S10 | Quantification of cortical actin on the apical side of the plasma membrane in gene-knocked down cells.

(A) The representative images of xz projections of cells with fluorescently labeled actin filaments (white) and nuclei (blue), which are also shown in **Figure 5c**. These plots demonstrate the corresponding profiles of the normalized fluorescence intensity along the apical side of the plasma membrane. **(B)** Quantification of cortical actin-based on the measured fluorescence intensities. The fluorescence intensities are normalized and presented as an arbitrary unit (a.u.). The number of projections analyzed is labeled on each bar. All statistical analysis was performed using the unpaired t-test. The asterisk represents the level of statistical significance for t-test: * $p < 0.05$, *** $p < 0.001$. The error bar represents the standard error of the mean.

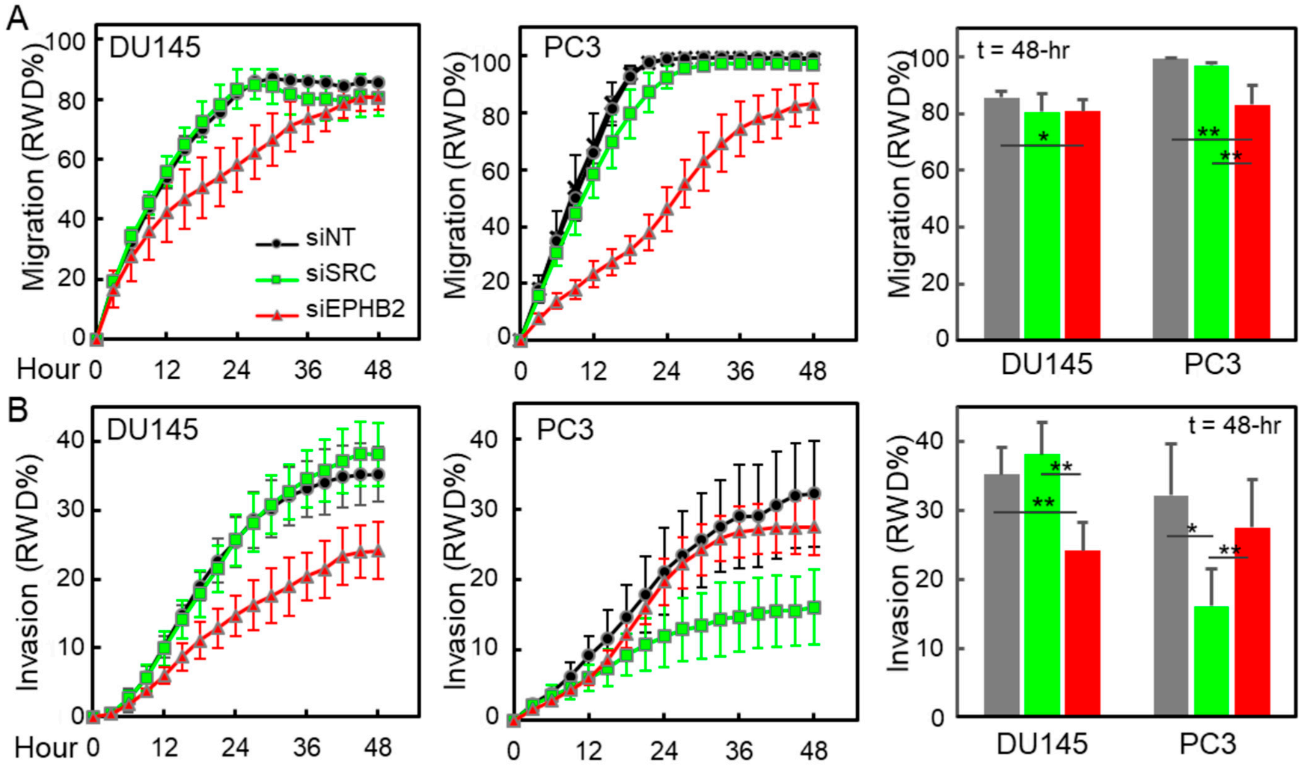

Figure S11 | Knockdowns of SRC and EPHB2 mRNA affect migration and invasion.

(A), (B) The imaging-based assays allow us to conduct the time-lapse analysis of cell migration and invasion of the siRNA-treated cells. The error bar represents the standard deviation. All statistical analysis was performed using the unpaired t-test. The asterisk represents the level of statistical significance for t-test: ** $p < 0.01$, * $p < 0.05$.

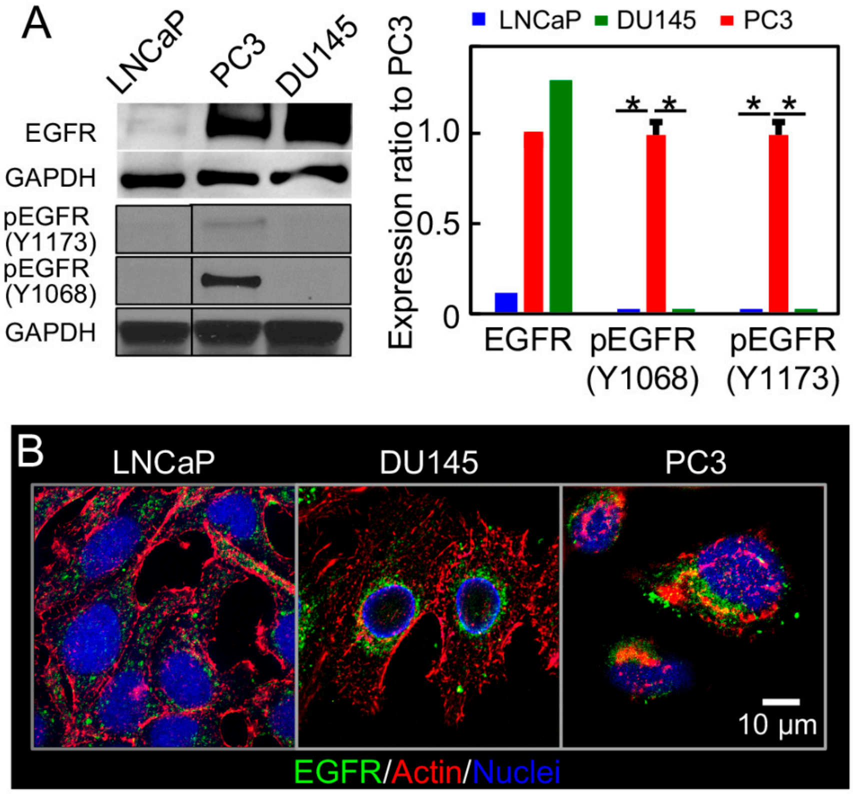

Figure S12 | EGFR expression of prostate cancer cell lines

(A) EGFR expression and phosphorylation of prostate cancer cell lines. **(B)** Immunocytochemistry images of cells fluorescently labeled with Anti-EGFR Affibody® Molecule FITC, the Alexa Fluor^{TM} 633 Phalloidin, and the Hoechst 33258.

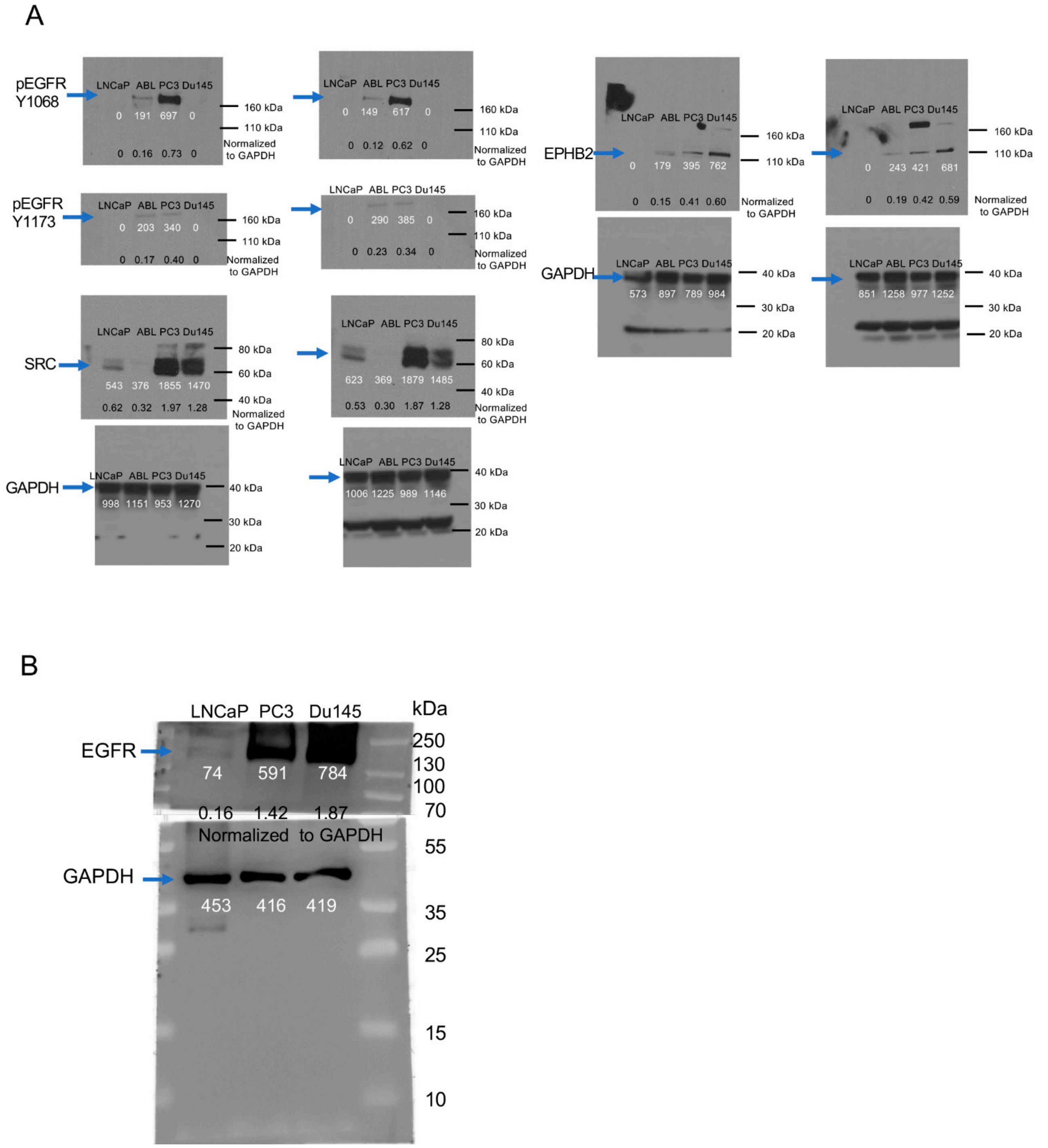

Figure S13 | Western blots

Western blot evaluation for **(A)** phosphorylated EGFR, EPHB2, SRC and **(B)** total EGFR. The duplicate experiments are shown side by side. The specific bands for those proteins are indicated by blue arrows. The molecular weight markers are labeled on the right sides.

Table S1 | Differentially expressed genes of EPH, EPHB forward, and SRC pathways in PC3.

**EPH-Ephrin signaling**			
Gene Name	*Up (+) or down (-) regulation in PC3*	*entrez-gene ID*	*entrez-gene name*
ARPC2#	+	10109	ARPC2 : actin related protein 2/3 complex, subunit 2, 34kDa
EPHB2*	+	2048	EPHB2 : EPH receptor B2
MYL9#	+	10398	MYL9 : myosin, light chain 9, regulatory
MYL12A*	+	10627	MYL12A : myosin, light chain 12A, regulatory, non-sarcomeric
FYN*#	+	2534	FYN : FYN proto-oncogene, Src family tyrosine kinase
EFNB1*#	+	1947	EFNB1 : ephrin-B1
PTK2*	+	5747	PTK2 : protein tyrosine kinase 2
EFNB3*#	+	1949	EFNB3 : ephrin-B3
EPHA2*#	+	1969	EPHA2 : EPH receptor A2
MYL6	+	4637	MYL6 : myosin, light chain 6, alkali, smooth muscle and non-muscle
RAC1*	+	5879	RAC1 : ras-related C3 botulinum toxin substrate 1 (rho family, small GTP binding protein Rac1)
CFL1*	+	1072	CFL1 : cofilin 1 (non-muscle)
ROCK2*	+	9475	ROCK2 : Rho-associated, coiled-coil containing protein kinase 2
SHB*	+	6461	SHB : Src homology 2 domain containing adaptor protein B
TIAM1*	+	7074	TIAM1 : T-cell lymphoma invasion and metastasis 1
ARHGEF28	+	64283	ARHGEF28 : Rho guanine nucleotide exchange factor (GEF) 28
ARPC1A	+	10552	ARPC1A : actin related protein 2/3 complex, subunit 1A, 41kDa
EPHA1*	-	2041	EPHA1 : EPH receptor A1
MYH14	-	79784	MYH14 : myosin, heavy chain 14, non-muscle
MYH10	-	4628	MYH10 : myosin, heavy chain 10, non-muscle
**EPHB forward signaling**			
Gene Name	*Up (+) or down (-) regulation in PC3*	*entrez-gene ID*	*entrez-gene name*
EPHB2**	+	2048	EPHB2 : EPH receptor B2
MAPK3	+	5595	MAPK3 : mitogen-activated protein kinase 3
PTK2**	+	5747	PTK2 : protein tyrosine kinase 2
PXN	+	5829	PXN : paxillin

RAC1**	+	5879	RAC1 : ras-related C3 botulinum toxin substrate 1 (rho family, small GTP binding protein Rac1)
RAP1B	+	5908	RAP1B : RAP1B, member of RAS oncogene family
GRB2	+	2885	GRB2 : growth factor receptor-bound protein 2
EFNB3**	+	1949	EFNB3 : ephrin-B3
EFNB1**	+	1947	EFNB1 : ephrin-B1
MAP4K4	+	9448	MAP4K4 : mitogen-activated protein kinase kinase kinase kinase 4
PIK3R1	-	5295	PIK3R1 : phosphoinositide-3-kinase, regulatory subunit 1 (alpha)
KRAS	-	3845	KRAS : Kirsten rat sarcoma viral oncogene homolog
**SRC signaling**			
Gene Name	*Up (+) or down (-) regulation in PC3*	*entrez-gene ID*	*entrez-gene name*
EPHB2	+	2048	EPHB2 : EPH receptor B2
SPRY2	+	10253	SPRY2 : sprouty RTK signaling antagonist 2
EYA4	+	2070	EYA4 : EYA transcriptional coactivator and phosphatase 4
ETS1	+	2113	ETS1 : v-ets avian erythroblastosis virus E26 oncogene homolog 1
ETS2	+	2114	ETS2 : v-ets avian erythroblastosis virus E26 oncogene homolog 2
ACTN1	+	87	ACTN1 : actinin, alpha 1
RPS27A	+	6233	RPS27A : ribosomal protein S27a
RIN3	+	79890	RIN3 : Ras and Rab interactor 3
MET	+	4233	MET : MET proto-oncogene, receptor tyrosine kinase
FGF1	+	2246	FGF1 : fibroblast growth factor 1 (acidic)
PALM2-AKAP2	+	445815	PALM2-AKAP2 : PALM2-AKAP2 readthrough
FGFR1	+	2260	FGFR1 : fibroblast growth factor receptor 1
SDC4	+	6385	SDC4 : syndecan 4
FLNA	+	2316	FLNA : filamin A, alpha
ANXA1	+	301	ANXA1 : annexin A1
ANXA2	+	302	ANXA2 : annexin A2
ANXA7	+	310	ANXA7 : annexin A7
CNTNAP1	+	8506	CNTNAP1 : contactin associated protein 1
ABLIM3	+	22885	ABLIM3 : actin binding LIM protein family, member 3
ARAF	+	369	ARAF : A-Raf proto-oncogene, serine/threonine kinase
MST1R	+	4486	MST1R : macrophage stimulating 1 receptor
ARHGDIA	+	396	ARHGDIA : Rho GDP dissociation inhibitor (GDI) alpha
ARHGDIB	+	397	ARHGDIB : Rho GDP dissociation inhibitor (GDI) beta
WNK4	+	65266	WNK4 : WNK lysine deficient protein kinase 4

FYN	+	2534	FYN : FYN proto-oncogene, Src family tyrosine kinase
ZYX	+	7791	ZYX : zyxin
AXL	+	558	AXL : AXL receptor tyrosine kinase
SPTBN1	+	6711	SPTBN1 : spectrin, beta, non-erythrocytic 1
TFG	+	10342	TFG : TRK-fused gene
TNFRSF11A	+	8792	TNFRSF11A : tumor necrosis factor receptor superfamily, member 11a, NFKB activator
F2R	+	2149	F2R : coagulation factor II (thrombin) receptor
EPB41L3	+	23136	EPB41L3 : erythrocyte membrane protein band 4.1-like 3
IQGAP1	+	8826	IQGAP1 : IQ motif containing GTPase activating protein 1
NEDD4	+	4734	NEDD4 : neural precursor cell expressed, developmentally down-regulated 4, E3 ubiquitin protein ligase
GNAI2	+	2771	GNAI2 : guanine nucleotide binding protein (G protein), alpha inhibiting activity polypeptide 2
NMT1	+	4836	NMT1 : N-myristoyltransferase 1
KRT19	+	3880	KRT19 : keratin 19, type I
PTTG1IP	+	754	PTTG1IP : pituitary tumor-transforming 1 interacting protein
GPC1	+	2817	GPC1 : glypican 1
TNS3	+	64759	TNS3 : tensin 3
STAT6	+	6778	STAT6 : signal transducer and activator of transcription 6, interleukin-4 induced
MMP14	+	4323	MMP14 : matrix metallopeptidase 14 (membrane-inserted)
CAPG	+	822	CAPG : capping protein (actin filament), gelsolin-like
CAPN2	+	824	CAPN2 : calpain 2, (m/II) large subunit
GRB2	+	2885	GRB2 : growth factor receptor-bound protein 2
CAV1	+	857	CAV1 : caveolin 1, caveolae protein, 22kDa
NR3C1	+	2908	NR3C1 : nuclear receptor subfamily 3, group C, member 1 (glucocorticoid receptor)
TGFBR2	+	7048	TGFBR2 : transforming growth factor, beta receptor II (70/80kDa)
TGM2	+	7052	TGM2 : transglutaminase 2
AFAP1	+	60312	AFAP1 : actin filament associated protein 1
PVRL3	+	25945	PVRL3 : poliovirus receptor-related 3
TIAM1	+	7074	TIAM1 : T-cell lymphoma invasion and metastasis 1
LAMC2	+	3918	LAMC2 : laminin, gamma 2
P2RY2	+	5029	P2RY2 : purinergic receptor P2Y, G-protein coupled, 2
KITLG	+	4254	KITLG : KIT ligand
CD44	+	960	CD44 : CD44 molecule (Indian blood group)
CD47	+	961	CD47 : CD47 molecule
H3F3B	+	3021	H3F3B : H3 histone, family 3B (H3.3B)
AKAP2	+	11217	AKAP2 : A kinase (PRKA) anchor protein 2
CDH2	+	1000	CDH2 : cadherin 2, type 1, N-cadherin (neuronal)
HIF1A	+	3091	HIF1A : hypoxia inducible factor 1, alpha subunit (basic helix-loop-helix transcription factor)

CAV2	+	858	CAV2 : caveolin 2
TRIP6	+	7205	TRIP6 : thyroid hormone receptor interactor 6
CFL1	+	1072	CFL1 : cofilin 1 (non-muscle)
GNG2	+	54331	GNG2 : guanine nucleotide binding protein (G protein), gamma 2
PFN1	+	5216	PFN1 : profilin 1
TRIP10	+	9322	TRIP10 : thyroid hormone receptor interactor 10
CHRNB1	+	1140	CHRNB1 : cholinergic receptor, nicotinic, beta 1 (muscle)
TXK	+	7294	TXK : TXK tyrosine kinase
PRKD3	+	23683	PRKD3 : protein kinase D3
CNN3	+	1266	CNN3 : calponin 3, acidic
PLA2G4A	+	5321	PLA2G4A : phospholipase A2, group IVA (cytosolic, calcium-dependent)
PLAUR	+	5329	PLAUR : plasminogen activator, urokinase receptor
PLAU	+	5328	PLAU : plasminogen activator, urokinase
HSPA2	+	3306	HSPA2 : heat shock 70kDa protein 2
PLSCR1	+	5359	PLSCR1 : phospholipid scramblase 1
ARHGEF6	+	9459	ARHGEF6 : Rac/Cdc42 guanine nucleotide exchange factor (GEF) 6
VCL	+	7414	VCL : vinculin
ROCK2	+	9475	ROCK2 : Rho-associated, coiled-coil containing protein kinase 2
EZR	+	7430	EZR : ezrin
SHB	+	6461	SHB : Src homology 2 domain containing adaptor protein B
TNC	+	3371	TNC : tenascin C
POLR2C	+	5432	POLR2C : polymerase (RNA) II (DNA directed) polypeptide C, 33kDa
POLR2L	+	5441	POLR2L : polymerase (RNA) II (DNA directed) polypeptide L, 7.6kDa
CDCP1	+	64866	CDCP1 : CUB domain containing protein 1
DAB2	+	1601	DAB2 : Dab, mitogen-responsive phosphoprotein, homolog 2 (Drosophila)
PPP2R5E	+	5529	PPP2R5E : protein phosphatase 2, regulatory subunit B,, epsilon isoform
CNN2	+	1265	CNN2 : calponin 2
SH3GLB1	+	51100	SH3GLB1 : SH3-domain GRB2-like endophilin B1
EFNB3	+	1949	EFNB3 : ephrin-B3
PRKCA	+	5578	PRKCA : protein kinase C, alpha
ARHGEF4	+	50649	ARHGEF4 : Rho guanine nucleotide exchange factor (GEF) 4
MAPK3	+	5595	MAPK3 : mitogen-activated protein kinase 3
IL6ST	+	3572	IL6ST : interleukin 6 signal transducer
GJA1	+	2697	GJA1 : gap junction protein, alpha 1, 43kDa
PHF19	+	26147	PHF19 : PHD finger protein 19

PAK6	+	56924	PAK6 : p21 protein (Cdc42/Rac)-activated kinase 6
SIRPA	+	140885	SIRPA : signal-regulatory protein alpha
ITGA2B	+	3674	ITGA2B : integrin, alpha 2b (platelet glycoprotein IIb of IIb/IIIa complex, antigen CD41)
HGS	+	9146	HGS : hepatocyte growth factor-regulated tyrosine kinase substrate
ITGB1	+	3688	ITGB1 : integrin, beta 1 (fibronectin receptor, beta polypeptide, antigen CD29 includes MDF2, MSK12)
PTK2	+	5747	PTK2 : protein tyrosine kinase 2
GAB2	+	9846	GAB2 : GRB2-associated binding protein 2
ASAP1	+	50807	ASAP1 : ArfGAP with SH3 domain, ankyrin repeat and PH domain 1
CCDC88A	+	55704	CCDC88A : coiled-coil domain containing 88A
CD59	+	966	CD59 : CD59 molecule, complement regulatory protein
CD63	+	967	CD63 : CD63 molecule
MYL12A	+	10627	MYL12A : myosin, light chain 12A, regulatory, non-sarcomeric
PXN	+	5829	PXN : paxillin
RAC1	+	5879	RAC1 : ras-related C3 botulinum toxin substrate 1 (rho family, small GTP binding protein Rac1)
SGK1	+	6446	SGK1 : serum/glucocorticoid regulated kinase 1
HBEGF	+	1839	HBEGF : heparin-binding EGF-like growth factor
DUSP4	+	1846	DUSP4 : dual specificity phosphatase 4
DUSP5	+	1847	DUSP5 : dual specificity phosphatase 5
DLGAP4	+	22839	DLGAP4 : discs, large (Drosophila) homolog-associated protein 4
LAMA5	+	3911	LAMA5 : laminin, alpha 5
LAMB1	+	3912	LAMB1 : laminin, beta 1
LAMB3	+	3914	LAMB3 : laminin, beta 3
SH3KBP1	+	30011	SH3KBP1 : SH3-domain kinase binding protein 1
ADAM12	+	8038	ADAM12 : ADAM metallopeptidase domain 12
S1PR3	+	1903	S1PR3 : sphingosine-1-phosphate receptor 3
PTP4A2	+	8073	PTP4A2 : protein tyrosine phosphatase type IVA, member 2
EFNB1	+	1947	EFNB1 : ephrin-B1
EPHA2	+	1969	EPHA2 : EPH receptor A2
SPRY4	+	81848	SPRY4 : sprouty RTK signaling antagonist 4
PTPRK	+	5796	PTPRK : protein tyrosine phosphatase, receptor type, K
ENO1	+	2023	ENO1 : enolase 1, (alpha)
FSD1	+	79187	FSD1 : fibronectin type III and SPRY domain containing 1
SMAD3	+	4088	SMAD3 : SMAD family member 3
ERBB3	-	2065	ERBB3 : erb-b2 receptor tyrosine kinase 3
EFS	-	10278	EFS : embryonal Fyn-associated substrate

SHROOM2	-	357	SHROOM2 : shroom family member 2
PTK2B	-	2185	PTK2B : protein tyrosine kinase 2 beta
OCLN	-	100506658	OCLN : occludin
PIK3R3	-	8503	PIK3R3 : phosphoinositide-3-kinase, regulatory subunit 3 (gamma)
CD2AP	-	23607	CD2AP : CD2-associated protein
KLK3	-	354	KLK3 : kallikrein-related peptidase 3
AR	-	367	AR : androgen receptor
IGF1	-	3479	IGF1 : insulin-like growth factor 1 (somatomedin C)
SLC9A2	-	6549	SLC9A2 : solute carrier family 9, subfamily A (NHE2, cation proton antiporter 2), member 2
CENPV	-	201161	CENPV : centromere protein V
FRMPD2	-	143162	FRMPD2 : FERM and PDZ domain containing 2
STAT3	-	6774	STAT3 : signal transducer and activator of transcription 3 (acute-phase response factor)
STAT5B	-	6777	STAT5B : signal transducer and activator of transcription 5B
EPM2A	-	7957	EPM2A : epilepsy, progressive myoclonus type 2A, Lafora disease (laforin)
FUBP1	-	8880	FUBP1 : far upstream element (FUSE) binding protein 1
GRIK2	-	2898	GRIK2 : glutamate receptor, ionotropic, kainate 2
THRB	-	7068	THRB : thyroid hormone receptor, beta
NANS	-	54187	NANS : N-acetylneuraminic acid synthase
CDH1	-	999	CDH1 : cadherin 1, type 1
GNB2L1	-	10399	GNB2L1 : guanine nucleotide binding protein (G protein), beta polypeptide 2-like 1
CDKN1B	-	1027	CDKN1B : cyclin-dependent kinase inhibitor 1B (p27, Kip1)
TUB	-	7275	TUB : tubby bipartite transcription factor
PIK3R1	-	5295	PIK3R1 : phosphoinositide-3-kinase, regulatory subunit 1 (alpha)
TRPV6	-	55503	TRPV6 : transient receptor potential cation channel, subfamily V, member 6
SYTL2	-	54843	SYTL2 : synaptotagmin-like 2
UTRN	-	7402	UTRN : utrophin
PRKACB	-	5567	PRKACB : protein kinase, cAMP-dependent, catalytic, beta
PRKAR2B	-	5577	PRKAR2B : protein kinase, cAMP-dependent, regulatory, type II, beta
PRKD1	-	5587	PRKD1 : protein kinase D1
CTNNA1	-	1495	CTNNA1 : catenin (cadherin-associated protein), alpha 1, 102kDa
PTEN	-	5728	PTEN : phosphatase and tensin homolog
PTP4A1	-	7803	PTP4A1 : protein tyrosine phosphatase type IVA, member 1

JUP	-	3728	JUP : junction plakoglobin
STX17	-	55014	STX17 : syntaxin 17
KLK2	-	3817	KLK2 : kallikrein-related peptidase 2
KRAS	-	3845	KRAS : Kirsten rat sarcoma viral oncogene homolog
RAP1B	-	5908	RAP1B : RAP1B, member of RAS oncogene family
DUSP2	-	1844	DUSP2 : dual specificity phosphatase 2
LEPR	-	3953	LEPR : leptin receptor
EPHA1	-	2041	EPHA1 : EPH receptor A1

Table S2 | The qRT-PCR primer sequences for validation of DE gene expression

Primer Name	Sequence (5'-3')	Product Length
ARHGEF28_F	GTACGTTGACATCTCCCCCG	115
ARHGEF28_R	TGAGGGCGGTGTGAATTTGA	115
ARPC1A_F	GACCGCATTGTCACTTGTGG	114
ARPC1A_R	CACAAAAGTAGCTGCGCGAT	114
ARPC2_F	CTGTTCCCTCGTCACACCAA	141
ARPC2_R	GAAGTCAGACGTTTTCGCCC	141
AXIN2_F	GCTAGGAGTGCGTTCATGGT	121
AXIN2_R	TGTGTCAATGGTAGGGCACC	121
CTNNB1_F	GGAGGAAGGTCTGAGGAGCAG	148
CTNNB1_R	ATTGTCCACGCTGGATTTTCAA	148
EFNA1_F	AAGACCGCTGCTTGAGGTTGA	139
EFNA1_R	GCACTGTGACCGATGCTATGTA	139
EFNB1_F	GCTTATCCTGGCAATGGGGT	126
EFNB1_R	TACCGTGAGAGTGAGGGAGG	126
EFNB3_F	GGACAGAAATGGCCTGGGAA	131
EFNB3_R	CCCCCAGACTAAGCCACTTG	131
EPHA1_2_F	AATCAGGGTGCAAGGAGCAA	91
EPHA1_2_R	ACCGATAGCCTAGTCTGGCA	91
EPHA1_F	GTGAGGTGTTCCCAGTGTCA	140
EPHA1_R	TTGGCATAAGGTTCAAGGCCA	140
EPHB2_F	AGCCATAAAAAGCCCCACCA	150
EPHB2_R	TGGTACTCGGCTTCTGTCAT	150
Primer Name	Sequence (5'-3')	Product Length
FYN_R	ATACTTCCCCAAACTGCCCATT	135
FZD1_F	CACCAACAGCAAACAAGGGG	118
FZD1_R	AGGATTGGCACGAACTCCAC	118
FZD2_F	CTGGAGCACCCCTTCCACT	74
FZD2_R	CGCTCGCCCAGAAACTTGTA	74
ID1_F	GGCGGCATGCGTTCCT	101
ID1_R	ACGTAATTCCTCTTGCCCCC	101
LATS2_F	CTAACTGTCGGTGTGGGGAC	114
LATS2_R	CCTCGGGTGCGATGTAGTTT	114
MYH14_F	CAGCACCGTGTCTTATGGTG	128
MYH14_R	TGATGCGGATGAATTTGCCG	128
MYL12A_F	CAACAACAGGACTTAACCACCA	135
MYL12A_R	GAAGGCCTCTTTGAACTCCTGA	135
PTK2_F	TCCCCAGAGCTCCTCAAGAAT	149

Primer Name	Sequence (5'-3')	Product Length
RAC1_F	TGCATTGTTGTGCCGAGAAC	102
RAC1_R	GAGAGCAAGTGTCTGCACCT	102
ROCK2_F	ACGTGGAGAGCTTGCTGGATG	146
ROCK2_R	TTCTGCCTTCATCTGTAGACCTCTG	146
SERPINE1_F	TTGCAGGATGGAACTACGGG	100
SERPINE1_R	GTGGCAGGCAGTACAAGAGT	100
SMAD3_F	CTCTTCTCTTCGCCGTGGGA	124
SMAD3_R	GCCCAAACTTCGCCTCAACT	124
SNAI2_F	ACAGCGAACTGGACACACAT	130
SNAI2_R	GCGGTAGTCCACACAGTGAT	130
SRC_F	ATCACCAGACGGGAGTCAGA	98
SRC_R	CAGTAGGCACCTTTCGTGGT	98
TEAD1_F	TTCGCCACGTGTGTTTGTTC	100
TEAD1_R	ACGCTTACATCACAAGCCCA	100
TEAD2_F	TACATCAAGCTGAGAACGGGG	106
TEAD2_R	CCTTCAACTTGGACTGGATTTC	106
TGFB1_F	ACGGATCTCTCTCCGACCTG	111
TGFB1_R	GACCGGGGGTGTCTCAGTAT	111
TGFB2_F	GTTACAACACCCTCTGGCTCA	96
TGFB2_R	TCTGTAGAAAGTGGGCGGGA	96
TGFBR2_F	ACACCAGCAATCCTGACTTGT	80
TGFBR2_R	TATGGCAACTCCCAGTGGTG	80
TIAM1_F	CCCCCTCCAGGAAACTGAAC	119
TIAM1_R	GGGACATTCTTGTCGACGGT	119
VAV3_F	CATTTCTCACGGCCTGTTGTG	150
VAV3_R	TGATTCCTGTGGCCAATGCTA	150
YWHAB_F	TCCCAATGCTACACAACCAGAA	121
YWHAB_R	CTGGGAGTTCGACACAGTGG	121
ACTB_F	CATGTACGTTGCTATCCAGGC	249
ACTB_R	CTCCTTAATGTCACGCACGAT	249

Table S3 | Summary of patients' pathological status and CTCs collected.

Patient #	Biochemical recurrence	CTC number collected and analyzed	Gleason score	PSA levels when CTCs were collected
VTC010	-	29	8	125.98
VTC004	-	18	7	6.31
VTC046	-	7	7	0.24
VTC034	+	19	8	1306.65
VTC048	+	6	Unknown	0.16
VTC035	+	12	8	0.5
VTC043	+	23	Unknown	3.48
VTC047	+	8	7	0.24
VTC027	+	14	7	37.17

Supplementary Reference

1. Arndt-Jovin, D.J., et al. *In vivo cell imaging with semiconductor quantum dots and noble metal nanodots*. in *Colloidal Quantum Dots for Biomedical Applications*. 2006. International Society for Optics and Photonics.
2. Dahan, M., et al., *Diffusion dynamics of glycine receptors revealed by single-quantum dot tracking.* Science, 2003. **302**(5644): p. 442-445.
3. Andrews, N.L., et al., *Actin restricts Fcϵ RI diffusion and facilitates antigen-induced receptor immobilization.* Nature cell biology, 2008. **10**(8): p. 955-963.
4. Hendriks, C.L., et al., *DIPimage: a scientific image processing toolbox for MATLAB.* Quantitative Imaging Group, Faculty of Applied Sciences, Delft University of Technology, Delft, The Netherlands, 1999.
5. Saxton, M.J., *Lateral Diffusion in an Archipelago - Single-Particle Diffusion.* Biophysical Journal, 1993. **64**(6): p. 1766-1780.
6. Martin, D.S., M.B. Forstner, and J.A. Käs, *Apparent subdiffusion inherent to single particle tracking.* Biophysical Journal, 2002. **83**(4): p. 2109-2117.
7. Saxton, M.J. and K. Jacobson, *Single-particle tracking: applications to membrane dynamics.* Annual Review of Biophysics and Biomolecular Structure, 1997. **26**(1): p. 373-399.
8. Daumas, F., et al., *Confined diffusion without fences of a G-protein-coupled receptor as revealed by single particle tracking.* Biophysical Journal, 2003. **84**(1): p. 356-366.
9. Di Rienzo, C., et al., *Fast spatiotemporal correlation spectroscopy to determine protein lateral diffusion laws in live cell membranes.* Proceedings of the National Academy of Sciences, 2013. **110**(30): p. 12307-12312.
10. Kusumi, A., Y. Sako, and M. Yamamoto, *Confined lateral diffusion of membrane receptors as studied by single particle tracking (nanovid microscopy). Effects of calcium-induced differentiation in cultured epithelial cells.* Biophysical Journal, 1993. **65**(5): p. 2021-2040.
11. Perillo, E., et al. *Single particle tracking through highly scattering media with multiplexed two-photon excitation*. in *SPIE BiOS*. 2015. International Society for Optics and Photonics.
12. Liu, C., et al., *Improving z-tracking accuracy in the two-photon single-particle tracking microscope.* Applied physics letters, 2015. **107**(15): p. 153701.
13. Perillo, E.P., et al., *Deep and high-resolution three-dimensional tracking of single particles using nonlinear and multiplexed illumination.* Nature communications, 2015. **6**.
14. Liu, Y.-L., et al., *Segmentation of 3D Trajectories Acquired by TSUNAMI Microscope: An Application to EGFR Trafficking.* Biophysical Journal, 2016. **111**(10): p. 2214-2227.
15. Le, V., et al., *Syndecan-1 in mechanosensing of nanotopological cues in engineered materials.* Biomaterials, 2018. **155**: p. 13-24.
16. Picco, A., et al., *Visualizing the functional architecture of the endocytic machinery.* Elife, 2015. **4**: p. e04535.
17. Li, C.-W., et al., *Eradication of triple-negative breast cancer cells by targeting glycosylated PD-L1.* Cancer Cell, 2018. **33**(2): p. 187-201. e10.
